# 3D Printing of Scaffold for Cells Delivery: Advances in Skin Tissue Engineering

**DOI:** 10.3390/polym8010019

**Published:** 2016-01-16

**Authors:** Deepti Singh, Dolly Singh, Sung Soo Han

**Affiliations:** 1 Biomaterials Lab, Department of Nano, Medical & Polymer Materials, College of Engineering, Yeungnam University, 280 Daehak-ko, Gyeongsan, Gyeongsanbukdo 712-749, Korea; dolly.ms@gmail.com; 2 Department of Surgery, Yale School of Medicine, Yale University, New Haven, CT-06510, CT, USA; deepti.singh@yale.edu

**Keywords:** 3D bioprinting, biomaterials, cell-matrix interaction, laser assisted bioprinting, stem cells

## Abstract

Injury or damage to tissue and organs is a major health problem, resulting in about half of the world’s annual healthcare expenditure every year. Advances in the fields of stem cells (SCs) and biomaterials processing have provided a tremendous leap for researchers to manipulate the dynamics between these two, and obtain a skin substitute that can completely heal the wounded areas. Although wound healing needs a coordinated interplay between cells, extracellular proteins and growth factors, the most important players in this process are the endogenous SCs, which activate the repair cascade by recruiting cells from different sites. Extra cellular matrix (ECM) proteins are activated by these SCs, which in turn aid in cellular migrations and finally secretion of growth factors that can seal and heal the wounds. The interaction between ECM proteins and SCs helps the skin to sustain the rigors of everyday activity, and in an attempt to attain this level of functionality in artificial three-dimensional (3D) constructs, tissue engineered biomaterials are fabricated using more advanced techniques such as bioprinting and laser assisted printing of the organs. This review provides a concise summary of the most recent advances that have been made in the area of polymer bio-fabrication using 3D bio printing used for encapsulating stem cells for skin regeneration. The focus of this review is to describe, in detail, the role of 3D architecture and arrangement of cells within this system that can heal wounds and aid in skin regeneration.

## 1. Introduction

**Figure 1:**
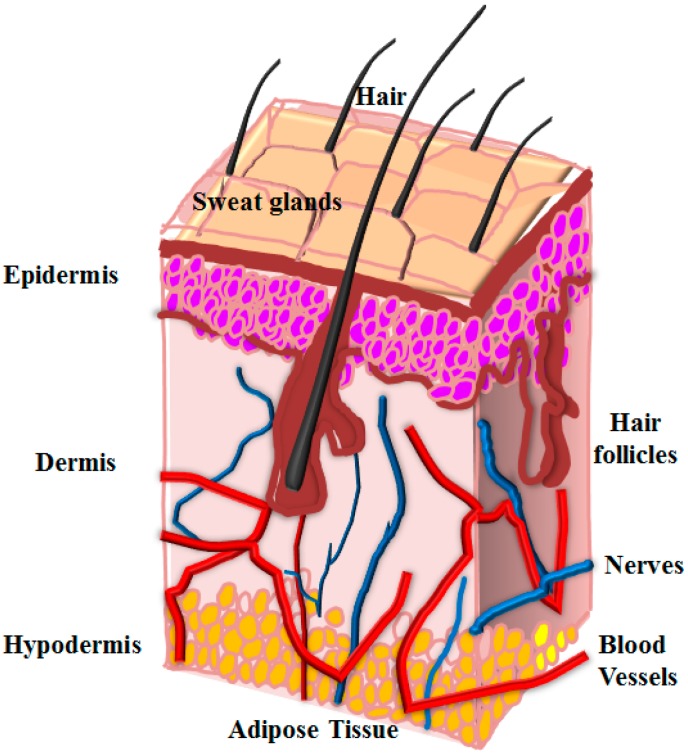
Schematic representation of different layers along with 3D architecture of skin.

The aim of engineering new biological material is that it can restore or replace a damaged or diseased tissue and organs [1,2,3]. The two most important components that decide the fate of tissue engineered construct are cells and artificial extra cellular matrices (ECMs), also known as scaffold or biomaterials that support cellular growth, differentiation, and migration [4,5,6,7]. Creating a construct by blending the principles of life sciences, developmental biology, and engineering that can address clinical problems, has been the focus of all researchers working in the area of regenerative medicine [8,9,10,11,12]. The foundation of the current research efforts in the field of tissue engineering is to recapitulate development processes that occur *in vivo* in clinical scenarios. This could be achieved with increased understanding of the roles of scaffold, stem cells, and signaling interaction of cells with artificial ECM [13,14,15,16,17]. However, before aiming to repair any tissue or organ, understanding of its anatomical structure and biogenesis is critical as it allows the users to control the conditions that could affect the neo-tissue formation. In the case of skin, a fundamental understanding of structure and functional relationship between normal and pathological tissue is required. Skin is the largest organ in human beings consisting of three different layers, with each of these layers playing significantly critical roles, as a unit act as a barrier, thermo-regulator, and cushion to internal organs from outer environmental conditions [18,19,20,21]. The epidermal layer is avascular, consisting of 95% keratinocytes, and covers the dermal layer (Figure 1) that is permeated by a complex nervous and vascular network. Beside this, there is a hypodermis layer that is primarily made up of loose connective tissues and lipid moieties, which helps in thermo-regulation of the system. Furthermore, in each of these layers there are different types of cells such as stem cells and epidermal basal cells (found in the basal layer) that are responsible for the continuous regeneration of epidermis. The dermis layer is again divided into the upper papillary layer, which is made up of thin collagen fibers, and a thick reticular layer that consists of thicker collagen fibers running parallel to the surface of skin [21,22,23,24,25]. Along with this collagen, elastin and reticular fibers are also found in this layer, and fibroblast is found to be the main cellular component that constantly secretes collagen and proteoglycans. Other types of cells found in skin are melanocyte that is responsible for pigmentation and protection against UV radiation, and Langerhans cells that act as the first line of defense against invading microbes [26].

In tissue engineering, cells play a critical role in skin regeneration. It is the interaction between cells, microenvironment, and molecular signals with the ECM that has made tissue engineering a reality. There are different lineages from which these cells can be derived, such as progenitor cells from bone marrow and adipose tissue to more differentiated local or systemic somatic cells. Although different research groups have worked on a diverse range of cells for creating an artificial skin substitute, the most commonly used cells are fibroblast, keratinocytes, hair-follicle associated cells, adipocytes derived stem cells, and melanocytes [27,28,29,30,31,32,33]. Tissue engineered scaffold are fabricated using different techniques to be architecturally as close to natural ECM as possible because even the simplest structure in our body is composed of highly organized ECM that facilitates the biological functioning of tissue. Engineering these complex tissues requires an interdisciplinary approach, which combines the material science innovation with cell biology development and growth factor chemistry by exploring the steps involved in organ development and morphogenesis [33,34,35,36]. As our knowledge of biology increases, the limitation of conventional methods used for creating tissue engineered scaffold become evident; the most apparent one being the importance of cell placement in the tissue construct as demonstrated by the complex spatial interplay observed between ECM and its cells [37]. Seeding cells at a specific location using spraying or manually is not precise; furthermore, ECM component and placement of growth factors are equally important, hence a new methodology for engineering a biological structure that can provide this high spatial resolution in neo-tissue is being developed [38,39,40,41,42,43]. Bio-fabrication strives to combine both the living and non-living parts of biological structure, but in a controlled fashion, and the emergence of 3D bioprinting has made it possible to achieve this precision in spatial resolution. The idea of placing small dots of cells in a specific location is the foundation of modern inkjet and laser printing methodology and over the years the size of dots has considerably reduced as innovations keep improving the technology [44,45].

## 2. Overview of Bio-Printing

3D bioprinting is used for engineering biological constructs, and usually involves dispensing cell onto a biocompatible matrix using sequential layer-by-layer technique to generate a tissue-like 3D structure. Some of the techniques used for 3D printing of cells include magnetic bioprinting, sterolithography, photolithography, and direct cell extrusion. Depending upon the organ to be printed, local cell population is isolated and cultured to attain the desired cell number. These cells are further mixed with a special liquefied medium that provides nutrition and oxygen required for keeping cells alive. Cell suspension is placed inside a tubular-like fusion for extrusion. This mix is placed in a cartridge and the structure is printed based on the medical scan of the patient. This pre-tissue is incubated and a bioreactor ensures a connective flow of nutrients for maturation of this construct into functional tissue. Encapsulating cells onto biomaterials during bioprinting provides these cells with structural support and enhances the maturation process.

### 2.1. Bioprinting of Hydrogels

**Figure 2:**
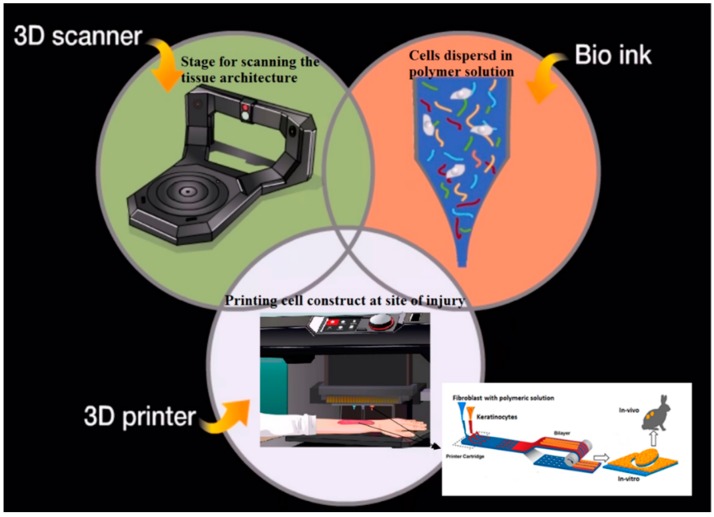
This diagram shows how the PrintAlive Bioprinter works for skin regeneration. Fibroblasts and keratinocytes two types of characteristic skin cells are kept in separate channels and spurted out of the 3D printer to custom an organized, multi-layer gel modified from [51].

Bioprinting is a computer-aided-design (CAD) based technique, wherein deposition of cells and scaffold material (like hydrogel) is achieved by a printer dispending method in a temporal and spatial controlled fashion (Figure 2). This technique has gained a lot of importance recently for engineering porous scaffolds, particularly the way cells are seeded during the printing stage onto the construct [44]. A complex 3D microenvironment can be generated by using a bioprinting system, in which the cells are integrated into hydrogel to mimic natural ECM of a particular tissue; thereby overcoming various challenges faced in tissue engineering and regenerative medicine [45]. One of the most used techniques is a drop on demand, in which thermal inkjet generates bubbles in the ink that force the ink to drop through the orifice of the microfluidic chamber; this "ink" is nothing but the combination of cell medium and materials that are to be printed. The ultimate resolution that could be attained using this technique directly depends on the print-head used for fabrication in the minimum droplet size approach. This resolution alters further as the droplet spreads on the surface, hence the final resolution of the biprinted construct will be different from the initial drop size [46]. There are different types of print-heads available, however, pressure driven microvalve is the easiest in comparison to thermal and piezoelectric print-heads used for drop-on-demand bioprinters. Spatial resolution obtained from pressure driven is lower, but throughput is found to be higher than the other two techniques. The older version of print-heads released in the 1990s is capable of reaching resolution in obtaining single cells precision theoretically, although it varies with the specific model of inkjet and the size of output orifice [47]. The best way to overcome the challenges of desktop inkjet is by developing a bioprinter with a specialized setup. Lee *et al.*, modified the existing inkjet for bioprinting keratinocytes and fibroblast by microvalves and used it for neural structure fabrication [48], however, major advances have been made using this technique in the area of skin tissue engineering. Binder *et al.*, modified desktop inkjet to attain precision, by which a high throughput of delivering 500 cells per drop was achieved. Furthermore, by bioprinting of fibroblast and keratinocytes using this design they were able to overcome pitfalls linked with single-cell inkjet bioprinting [49]. This was a cartridge based system, which allowed any type of biomaterials or cells to be packed into a cartridge to be bioprinted at a precise location in the wound. This work provided a template to researchers who were seeking to alter the settings of desktop inkjet printers and showed that personalized modification could be done in order to produce next generation engineered complex functional grafts [50].

To further provide proof of concept for the above work, Binder *et al.*, set up an experiment in which they bioprinted skin by using fibroblast and keratinocytes for wound healing applications. During this experiment, two major facets of control delivery were tested. (1) Ability of the delivery system to achieve controlled delivery of two different types of cells which was performed by validating the movement system, controlling the software used for *in-situ* bioprinting and using a cartridge-based valve delivery system for the skin. (2) Usage of a fibrin/collagen type 1 delivery matrix that could act as scaffolding material for printed cells. In this experiment, Binder dissolved fibrinogen and collagen in equal quantities for thrombin bioprinting; the reason for using thrombin was for converting fibrinogen to fibrin which results in a liquid polymer solution to convert into a gel thus acting as a scaffolding system. This was a small-scale system which had two different cartridges for fibroblast and keratinocytes, with spatial resolution set up at 1.57 µm and pressure at 6.89 kPa. Cells were embedded into the matrix and after printing were implanted at the site of the wound. It was found that these cells remained at the exact site of the wound area and evaluation of lesion over six weeks showed the quick closure of the wound in test mice. This design provided a powerful tool that can augment the way burn cases are treated, since the cartridge based system can easily be transported to different patients. Furthermore, the *in-situ* skin printing technique ensures that cells remain at a precise location post transplantation; and combining cells and matrix with anti-microbial agent could possibly be a new treatment method providing skin coverage and functional outcome recovery; even in extensive burn cases [50].

### 2.2. Emergence of 3D Printing for Skin Repair

Although there are many conventional tissue engineering approaches which facilitated the plan, architecture, and design for first generation skin grafts, there are still many aspects that need improvement. In the last decade, printing techniques have drastically improved and moved from 2D to 3D, wherein an additional layer is distributed for three dimensional formations. 3D printing provides flexibility to control the geometry at the micro/nano-cellular level, which modulates precise cell-cell interaction in a 3D environment [52,53]. This automated system is on demand for “free-form” material fabrication that is similar to the complex architecture of living tissues and maintains high cell viability while dispensing soluble factors and phase-changing hydrogels in preferred patterns [54,55,56]. This approach could be extremely useful for skin tissue engineering, as it allows researchers to layout precisely a multi-layered matrix and cells in “layer-by-layer” assembly in turn bio-mimicking the skin tissue. Skin tissue is highly-stratified and multilayered, hence it becomes a perfect model for demonstrating the advantages and strengths of using 3D bioprinting to overcome the limitation of traditional tissue engineering techniques. The generation of bio-artificial skin using 3D printing technique requires isolation of autologous cells, which is then proliferated in the laboratory to obtain the desired cell numbers followed by layering of cells via a 3D bioprinter [56,57,58]. This step could either be by layering of cells on a scaffold or scaffold-free formation of cellular spheroids. If a scaffold is used, it generally is to provide structural support and is most often removed after cells fuse together to create their own support system [58]. There are different approaches through which 3D bioprinting can be achieved as proposed by Atala *et al.* [59].

#### 2.2.1. Biomimicry

This is a biologically inspired technique, in which the specific cellular functional component of organ or tissue; like the multi-layers of skin or branching of the vascular system is mimicked. For success of this approach, the organ has to be replicated at the micro-scale level. Its application in 3D printing includes identical reproduction of the cells and extra cellular component of the specified tissue or organ. This method requires complete understanding of the precise cellular arrangement and functionality along with knowledge of the micro-environment, different soluble or insoluble factors, and the nature of the biological forces in the targeted tissue, which are critical for tissue functionality. A thorough knowledge and collaboration of imaging, biomaterials, biophysics, engineering, medicine, and cell biology are required to achieve a significant breakthrough using this approach [60,61,62,63,64].

#### 2.2.2. Autonomous Self-Assembly

According to Atala *et al.* [59], with in-depth knowledge of the development process cascade and organogenesis, the skill for manipulating the microenvironment in a way that it compels the embryonic system to differentiate as bioprinted tissues; biological tissue could be reproduced by following the development map of the embryo. In the earliest stage of embryo formation, cellular moieties secrete their own ECM components with precise cell signaling, independent organization, and patterning which work together to produce biologically functional intrinsic micro-architecture. Cellular spheroids that undergo fusion and cellular reorganization to mimic architecture of the developing tissue is another version of the self-assemble approach. However, cellular spheroids are basically “scaffold-free” and are dependent upon the magnetic suspension technique. The spheroids formed can vary in size, depending upon the parameters set by the user. Their complete biological functionality directly depends upon the cells secreting their own ECM component, following the signaling pathways for histogenesis and the localization process [64,65,66,67,68].

#### 2.2.3. Mini-Tissue

This is another concept relevant to both types of approaches mentioned above and focuses on the functional unit of any organ or tissue, henceforth called “Mini-tissue”; for example, nerve cells being the smallest functional unit of the nervous system or nephrons for the kidney. These small units can be assembled together to form a larger construct using self-assembly or biomimicry techniques. Steps involved in this are production of small cellular spheroids that act as mini-tissue which are further stacked or assembled into macro-tissue by using bio-inspired design of organs [69,70,71,72,73,74,75].

**Figure 3:**
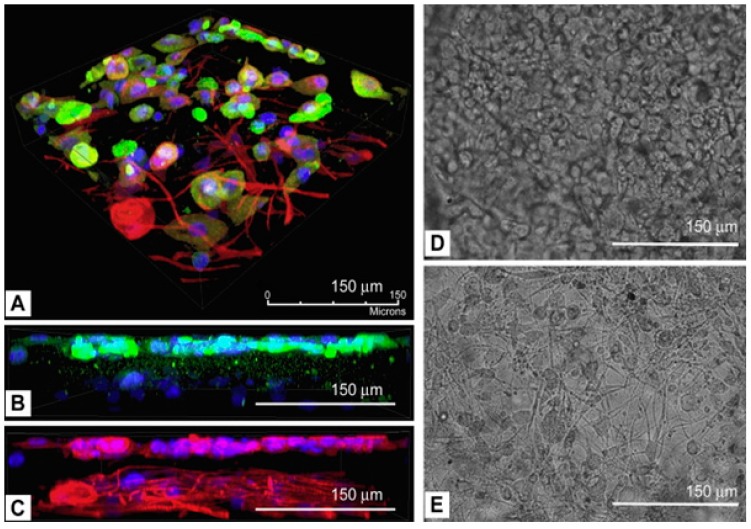
Confocal images of multi-layered printing of fibroblast and keratinocytes (FB and KC) on the cell culture dish. (**A**) Z stacking using immunofluorescent images showed interlayer distance ~75 μm was observed. (**B**,**C**) keratin-containing KC layer and β-tublin-containing KC and FB. Bright-field images on (**D**) KC layer and (**E**) FB layer also confirmed the immunohistochemistry findings. Reproduced from [77]. Copyright 2009 with permission from Elsevier.

Recent proof of concept study performed by Lee *et al.* [76] showed the usage of the 3D printing technique to engineer human skin in a layer-by-layer assembling process using fibroblast and keratinocytes cells. In this work, the authors used eight independently controlled cell-dispensing channels that could position cells, ECM scaffold material, and other growth factors in a user-defined 3D prototype. Each dispense was operated by electromechanical valves which were mounted onto a three-axis; *XYZ* robotic platform; with high precision and liquid solution was dispensed at the gate-opening stage of the micro valves by pneumatic pressure. Depending upon the viscosity of the materials, the minimum resolution can vary; for example, in the case of aqueous medium which include water and cell culture medium, the minimum resolution that could be obtained is ~100 µm; however, resolution is higher in the case of matrix proteins and collagen. This preliminary work gave a platform for potential use of 3D bioprinting for complex human skin, as the results suggested that 3D printed skin could maintain its original shape, architecture, physical dimensions, and structure throughout its culture time. This was in contrast to scaffold fabricated manually which showed significant shrinkage from the second day onwards [77]. These results suggest that 3D printing could be used for fabrication of a matrix with high dimensional control for repairing and regeneration of complex tissue like skin [76] (Figure 3). In another study, performed by Murphy *et al.*, hydrogels were fabricated by a bioprinting technique that acts as a substrate for cellular proliferation and migration of endogenous factors within this structure, which in turn actively promotes wound healing. A cartridge based-drug delivery scheme was employed, in which a laser scanning system was mounted onto a portable *XYZ* plotting bioprinter. Data was pieced together to form a wound surface model that was further filled completely by hydrogel dispensed by a delivery system. Single or combination of the techniques could be used to print a 3D complex biologically active, structurally similar, with mechanically stable tissues or organ [78]. However, to successfully obtain functional tissue, bioprinting imaging and material science is required as they play a critical role during testing of *in vitro* maturation of printed tissue. Polymers that can mimic the natural ECM component also provide a higher chance of successful tissue regeneration.

### 2.3. Laser Assisted Bioprinting

**Figure 4:**
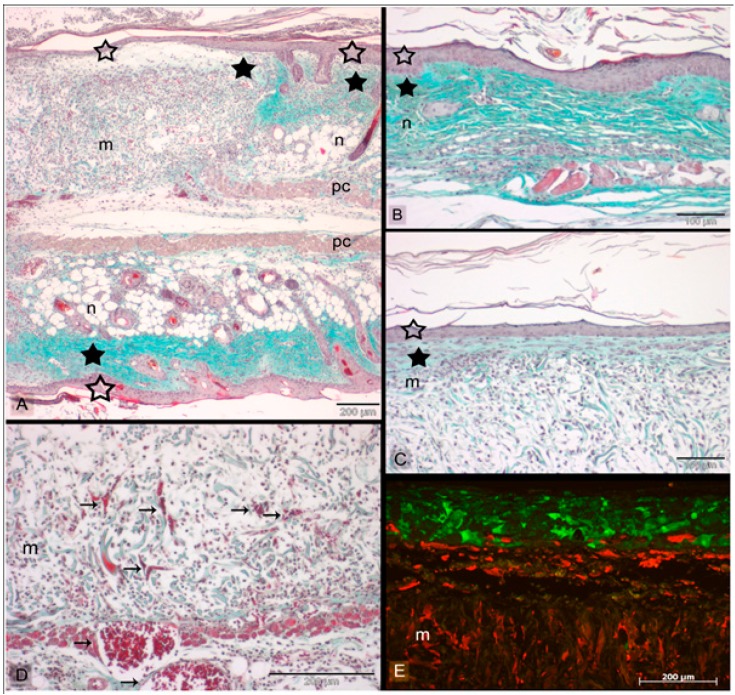
Skin grafts implanted in mice for 11 days in dorsal skin fold chambers. Sections are stained with Masson’s trichrome (**A**–**D**) and analyzed with fluorescence microscopy (**E**), respectively. (**A**) Shows the junction between the inserted skin graft (m = Matriderm^®^) and host mouse skin (n) at the wound edge after 11 days. The engineered and the intact skin part in the sandwiched skin model are separated by the panniculus carnosus (pc). Both in native mouse skin (**B**) and printed skin graft (**C**) a thick epidermis (empty asterisks) and corneal layer can be seen. In skin graft, the epidermis layer is formed by the printed keratinocytes (**E**) noted by green fluorescence that’s emitted by HaCaT-eGFP cells. The fibroblasts (NIH3T3 cells-mCherry) is seen partly migrating into the Matriderm^®^ (yellowish fibres). The fibroblasts that remains on top of the Matriderm^®^, exhibits an outstretched morphology (**C**), along with collagen deposition (filled asterisks). Blood vessels (arrows) can be noticed in the skin constructs (**D**). Scale bars depict 200 µm (**A**,**D**,**E**) and 100 µm (**B**,**C**) (Reprinted under open access distribution license from [79]).

Even though tissue engineering promises high potential for new skin production, there are limitations which need to be overcome. The challenge remains to engineer precise and complex hierarchy in new tissue, that allows different types of cells to co-exist and can arrange into a unique 3D pattern. Laser assisted bioprinting (LaBP) technique has emerged as a solution to the traditional scaffold fabrication approach, as it offers the opportunity to engineer high resolution 2D and 3D patterns by incorporating different cell lines. These could either be mouse endothelial cells, human osteosarcoma, rodent olfactory ensheathing cells, fibroblast and keratinocytes, rat Schwann and astroglial cells or cartilage and osteoblast cells. All of these have survived the transfer process without showing any damage or alteration in cellular phenotypes. Michael *et al.*, worked on fabrication of a multi-layer fully cellularized skin substitute for burn patients using LaBP techniques. The transplanted skin equivalent was tested on an animal model to confirm its ability to achieve cellular behavior, tissue formation, and differentiation of keratinocytes accompanied by neovascularization in dorsal skin fold chamber of mice. As skin is a multifaceted organ consisting of various types of cells with sub-structural arrangement in defined spatial configurations, LaBP is one of the best suited techniques for skin tissue engineered substitute as demonstrated by this work. The printed cells form tissue that is similar to native skin producing fibroblast assisted collagen, with dense epidermis and presumably supporting differentiation of keratinocytes. Even though it was a short study of less than 11 days to confirm keratinocytes differentiation, the proliferation marker (Ki67) was found at the supra-basal layers hinting at the beginning of keratinocytes differentiation (Figure 4). It is well known that keratinocytes found in stratum basale maintain their proliferation unlike in other layers where differentiation stops proliferation. Using this process leads to ingrowths and integration of skin graft into the wound. However, with thinner epidermis or absence of rete ridges, graft failure could occur; and LaBP provides the solution for printing these rete ridges and thick epidermis [79]. In another experiment performed by Koch *et al.*, laser printing based on “laser-induced forward transfer (LIFT)” was used as a new technique for skin tissue engineering. In this work, fibroblast/keratinocytes and MSCs (mesenchymal stem cells) were preferred due to their high self-renewal and easier application in regenerative medicine. The influence of using LIFT on these cells was evaluated by quantifying cell survival rate, DNA damage or modification of cell surface receptors/markers, proliferation, and apoptotic activity. It was found that 98% ± 1% fibroblast/keratinocytes and about 90% ± 10% hMSC were able to survive the transfer step. Furthermore, all three types of cells retained their ability to proliferate after LIFT, and there was no damage or change in DNA or surface marker, which was confirmed by fluorescent activated cell sorting (FACS) testing. This study was useful in demonstrating that LIFT could be potentially used as a suitable technique for generating the computer controlled arrangement of different cells into one functional tissue which could be further explored for engineering future generation tissue replacement other than skin [80].

## 3. Advances in Stem Cells for Skin Substitute

One area that has raised hopes of tissue engineered skin substitute is the progress that has been made in the field of stem cells and biomaterials, especially with the emergence of induced pluripotent stem cells that negate the concerns that were associated with embryonic stem cells.

### 3.1. Role of Induced Pluripotent (iPSCs) and Embryonic Stem Cells

**Figure 5:**
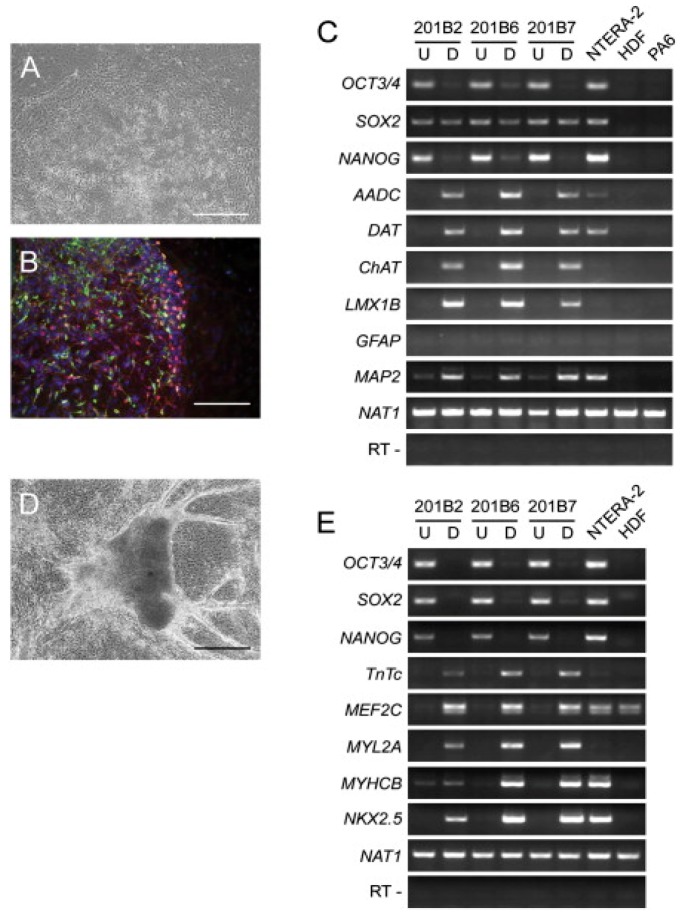
Directed differentiations of human induced pluripotent stem cells (iPS). (**A**) Phase-contrast image (**B**) Immunocytochemistry with βIII-tubulin (red) and tyrosine hydroxylase (green) and Hoechst 33342 nuclei (blue) antibodies of differentiated iPSCs cells after 18 days culture on PA6. (**C**,**E**) Reverse transcriptase polymer chain reaction (RT-PCR) analyses of dopaminergic neuron and cardiomyocyte markers. (**D**) Phase-contrast image of iPS cells differentiated into cardiomyocytes. (Bars = 200 μm (**A** and **D**) and 100 μm (**B**)). Reproduced from [82]. Copyright 2006 with permission from Elsevier.

iPSCs are the most recent advance made in the area of cell biology wherein reprogramming somatic cells provides an exciting alternative to the use of embryonic stem cells (ESC). Somatic stem cells are multipotent with self-renewal capacity, found in various organs such as brain, bone marrow, dermal tissue, and skeletal muscle that enable regeneration of damaged tissues [81]. However, they do not have unlimited proliferative ability thereby, limiting their application in the field of regenerative medicine. In order to overcome the limitations and concerns associated with ESCs’ use in the clinical arena, iPSCs were established in 2006 by Takahashi and Yamanaka [82] (Figure 5). This group used mouse fibroblast cells and introduced four transcription factors (Oct 3/4, c-MYC, SOX2, and Klf4) that are responsible for unlimited proliferative characteristics in ESCs. Retrovirus was used as transduction agent and once reprogrammed, cells were carefully chosen by expression of β-geo cassette. However, when injected into nude mice they failed to form adult chimeras and showed different gene expression along with altered DNA methylation patterns. Yet, this experiment opened a complete new area of research, and a year after, human iPSCs were established by introducing the same transcriptional factor on fibroblast cells along with another set of transcriptional factors (Oct 3/4, Nanog, Lin28, and SOX 2) [83]. These human iPSCs were phenotypic, genotypic, and epigenetic status of pluripotent expression (cell-specific genes) similar to human ESCs [84,85]. Furthermore, the cells were found to have the capacity to differentiate into all three germ layers and hence named as induced pluripotent stem cells. Establishing direct reprogramming somatic cells was a milestone in stem cells research, as it provided an invaluable source of unlimited cell supply for regenerative medicine [86]. iPSCs’ rapid progression also shows their vast implication in the field of biomedicine, and with high similarity to ESCs, iPSCs easily bypass the ethical controversy surrounding ESCs’ use in humans. They also offer superior advantage, such as direct programming of somatic cells, enabling patient-specific cells to be obtained that can be modulated for high pluripotency [87,88]. The chances of immune-rejection are also minimized, but the best advantage that iPSCs offer is the chance for correcting any genetic disorder in patients. For example, for patients suffering from Fanconi anemia, corrections can be made using lentiviral vector that carries coding for FANCA or FANCD2 and transduces into fibroblast. These corrected cells can further be expanded into iPSCs as effectively as any wild type fibroblast. Continuous change and improvement in iPSCs’ reprogramming paves the way for newer approaches especially in the case of skin tissue engineering. New techniques provide a way to produce iPSCs from keratinocytes that are isolated from hair follicles of adult patients and then using these cells for differentiating into various skin related cells [89].

Research performed by Bilousova *et al.*, shows that iPSCs can be differentiated in laboratory conditions into a range of skin-like cell lines with capacity to form multi-differentiated epidermis with hair follicles and sebaceous glands [90]. Skin serves as an ideal platform for the application of iPSCs because, unlike other tissues and organs, skin is easily accessible and cells can be isolated from both patient and healthy individual. Interestingly, it is not just accessibility that makes skin an ideal platform, it is also the ability of skin cells to give rise to iPSCs far more efficiently when transduced with “Yamanaka factor” than when compared to other fibroblast cells. A study performed by Utikal *et al.*, 2009 shows that “melanocytes and follicular dermal papilla cells” were able to upregulate SOX2, which happens along with Klf4 murine dermal papilla cells, and could easily differentiate into iPSCs [91]. Another study by Tsai *et al.*, shows that Oct4 alone was sufficient to induce pluripotency in somatic skin cells, indicating the possibility of a less laborious protocol for reprogramming cells into iPSCs [92]. The few experiments that have shown the proof of concept are the ones conducted by Itoh *et al.* [93] and Veraitch *et al.* [94] demonstrating that reprogramming keratinocytes to iPSCs results in regeneration of epidermal structures following exposure to BMP4 and retinoic acid. In another set of experiments, melanocytes were used for reprogramming and the iPSCs obtained were able to convert into embryoid bodies when incubated with WNT3A and endhothelin-3 stem cell factors [95]. For establishing an iPSC based cell therapy for skin regeneration, there are several issues that need to be addressed. Methodologies for *in vitro* proliferation and establishment of 3D construct; equivalent to normal skin in the sense of regeneration of epidermal and dermal layers using somatic cells; is already available for burn case patients. However, a major obstacle remains around whether the research performed using iPSC for regeneration of other tissue could be used to draw a parallel to. If so, then it will not be long before iPSCs’ based skin equivalent could be in clinical usage [96].

### 3.2. Manipulation of Mesenchymal Stem Cells for Skin Repair

MSCs are multipotent self-renewing cells that can differentiate into all lineages of connective tissue of mesenchymal origin. Besides this, MSCs also play a significant role in regulating the immune system, inflammation reactions as well as possessing a potent tissue protective and repairing mechanism. This makes them ideal for application in regenerative medicine, especially skin tissue repair. Skin represents a perfect model system, in which the role of progenitor cells as a source for regenerative medicine can be easily investigated, since keratinocytes are one of the few well-characterized examples of adult stem cells. Another advantage of using MSCs is that they can be derived from the patient, thereby removing any inherent problem associated with immune reaction and rejection. Furthermore, treating patients with their own cells also helps avoiding all ethical and moral objections related to stem cell usage. A potential source of MSCs is bone marrow, found circulating mostly in blood, which appears more fibroblastic and the cells rapidly enter injured tissue within a wound chamber model. Their existence in wound chamber has been thought to be mainly due to their recruitment ability from adjacent subcutaneous tissue. However, on closer investigation, this ability was attributed to peripheral blood, since the large number of fibroblast like cells described as CD34+/collagen+/vimentin+ (termed fibroblast due to its distinct phenotype), was directly proportional to the entry of circulating inflammatory cells and was not restricted to exclusively slow migration from adjacent connective tissue [96]. These cells; even though they constitute only 10% of all infiltrating cells; are thought to play a diverse role in skin repair, inducing angiogenesis and the wound healing process; both *in vitro* and *in vivo*. They produce chemo attractants which can recruit CD4+ cells along with expressing the receptor for CCR7, which is involved in cell migration into the wound process. However, the process and intercellular signaling mechanism that can modulate fibroblast proliferation, differentiation, and trafficking is only partially understood. As such, these cells have broad clinical application and different researchers have provided proof of this concept.

**Figure 6:**
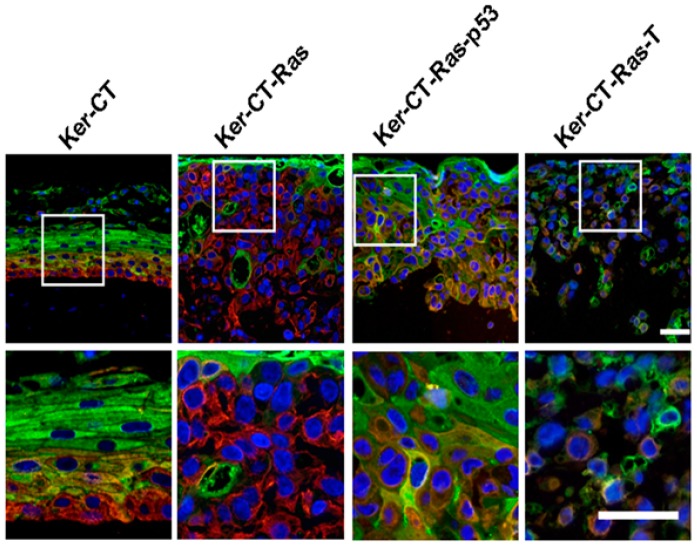
Double-staining performed in 21 days skin graft with keratin-14 red) and involucrin (green) stained the lowermost and uppermost layers respectively. Some evidence of co-localization (yellow to orange) in the intermediate layers was found. Both stains were found to be partially retained in Ker-CT-Ras keratinocytes, but were far less in Ker-CT-Ras-p53 keratinocytes. Scale bar: 40 µm. Reprinted under open access distribution license from [100].

**Figure 7:**
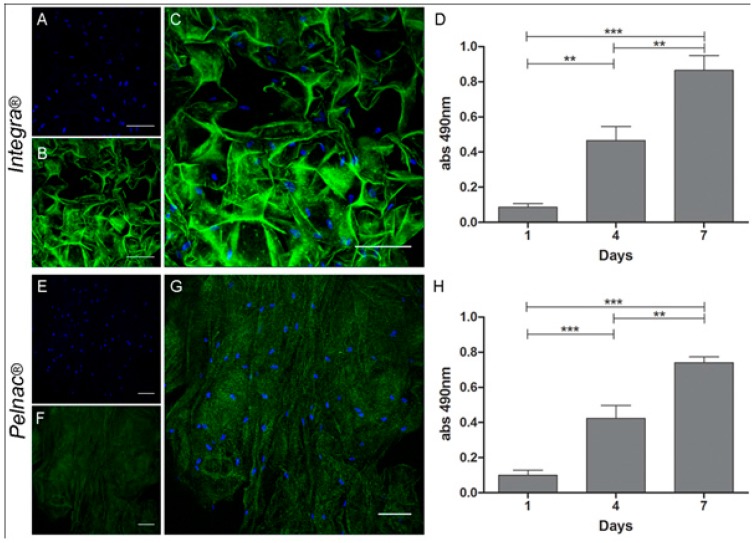
3D cultures of human SD-MSCs in different polymeric materials (**A**–**D**) Integra and (**E**–**H**) Pelnac. Confocal microscopy of SD-MSCs stained with nuclear staining (DAPI), (**B**,**F** and **C**,**G**) are dermal substitutes from each group (green autofluorescence) and merged images respectively. (**D**,**H**) MTS cell viability assay of SD-MSCs cultured in Integra and Pelnac, respectively. *** *p* < 0.001, ** *p* < 0.01. Reprinted under open access distribution license from [97].

In the quest of finding a new strategy for skin regeneration, Trentin *et al.* [97] provided effective treatment, especially in the case of cutaneous wound. Stromal MSCs (SD-MSCs) were cultured on 3D culture with dermal substitute (Integra and Pelnac) for one week and different tests were performed to check the outcome. It was found that when MSCs were cultured in a 2D system, they expressed MSCs’ characteristic markers such as fibronectin, CD105, and α-SMA along with markers for neural lineages like βIII-Tublin and Nestin. Similarly, Integra and Pelnac equally support SD-MSCs’ proliferation yet SD-MSCSs successfully maintained their multilineage markers (Figure 6). This successful study was able to demonstrate that dermal substitutes support MSCs’ proliferation, and it could be one way to effectively use MSCs in combination with dermal substrate for skin regeneration. Perng *et al.* used pNIPAAM polymer seeded with bone marrow derived from human mesenchymal cells to repair a skin defect in nude mice. They tested the level of human pan-keratin and cadherin, which are markers of epithelial regeneration and found that in test mice, the expression of both significantly increased and MSCs were able to differentiate to form epidermal cells, thereby repairing skin defects [98]. In an attempt to further progress skin regeneration, Sheng *et al.* successfully induced MSCs derived from bone marrow into sweat gland cells by co-culturing techniques [99]. Vaughan *et al.* showed that coordinated proliferation and stratification of keratinocytes could be achieved by altering the expression of H-ras and it is one of the important parameters for maintenance of artificial skin construct (Figure 7) [100]. Another problem associated with stem cells is that there is no universally accepted cell surface marker which can confirm that the phenotype and genotype of these cells have not changed due to some rigorous methodology involved in the process of regenerative medicine. However, even the autologous stem cells’ usage presents its own challenges; such as a complex isolation process, as well as very costly and time consuming, to generate enough stem cells for repairing or regenerating skin defects.

### 3.3. Adipose-Derived Stem Cells for Skin Regeneration

Adipose stem cells’ (ASCs) discovery opened a new era and made stem therapy a reality, since the cells can be harvested in large quantity and applied instantly without needing time for culture expansion. These progenitors, which are readily available due to the obesity epidemic and the cosmetic evolution (lipoaspiration), are known to secrete cytokines that promote fibroblast migration in the wound healing process. ASCs also up-regulate vascular-endothelial growth factor (VEGF) which enhances neovascularization [101]. Unlike MSCs, the surface markers for ASCs are clearly defined and only if CD29, CD73, and CD90 are expressed in consecutive generations, can these cells be used for cell therapy. Bone marrow fluid is known to contain a very small percentage of MSCs, whereas the amount of ASCs isolated is 500 fold greater from the corresponding weight of adipose tissue. It is relatively homogenous on surface immunophenotype and displays similar surface antigen; if not identical to MSCs; making it a potential candidate for regenerative medicine. Huang *et al.*, 2013 created an ulcer in the rat model by electron beam radiation and treated it with ASCs, followed by evaluating the wound healing assay. It was found that after three weeks of receiving regular ASCs, the wound size significantly reduced in comparison to control (*p* < 0.01). On histological testing of the edges of the wound and immunoblotting for checking the re-epithelialization area, both indicated that the group which received ASCs developed neo-angiogenesis and colocalized with endothelial cell markers in the wounded tissue. This proved that using ASCs could potentially accelerate the wound healing process. Epithelization is one of the most important steps for wound healing as it ensures new epithelium formation. Various groups have worked on proving the potential of ACSs for wound healing and skin regeneration [101].

**Figure 8:**
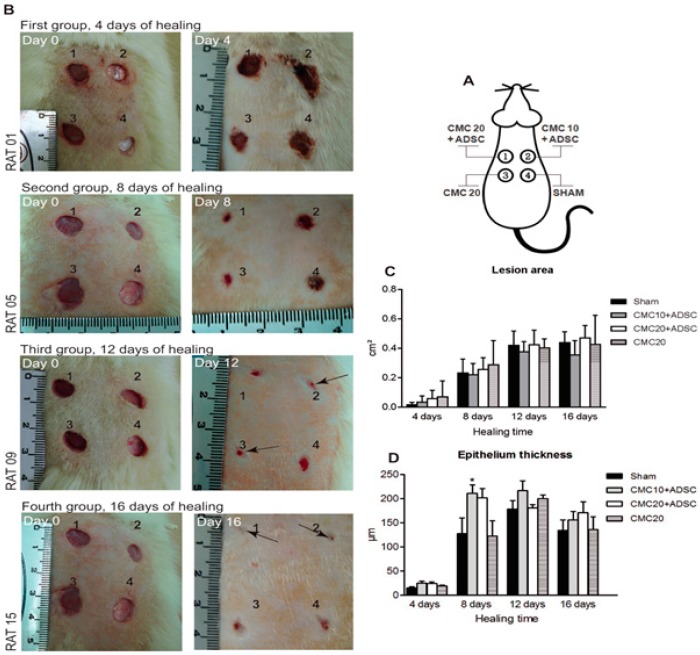
Progression of wound healing. (**A**) Wound model. Treated with four different approaches **upper left** (**1**) 100 µL of carboxymethylcellulose (CMC) 20 mg/mL + 26 × 10^6^ of adipose derived stem cells (ADSCs); **upper right** (**2**) 100 µL CMC 10 mg/mL + 26 × 10^6^ of ADSCs; **bottom left** (**3**) 100 µL of CMC 20 mg/mL; **bottom right** (**4**) Sham. (**B**) Animal, representative of each group showing wound closure, from day 0 to day 16. (**C**) Evaluation of the area of lesions and panel. (**D**) Determination of epithelium thickness. * *p* < 0.05. Reproduced under open access distribution license from [102].

Another study conducted by Wink *et al.*, 2014 showed that lesion with great loss of skin and a broad burn area when treated with heterologous skin grafts could lead to rejection. However, when combined with ASCs and carboxymethylcellulose (CMC) to treat skin lesion in the rat model, it showed promising results. The test group received 10 mg/mL concentration of CMC along with ASCs showing increased cell proliferation in granulation tissue and thick epithelium formation when compared to control (untreated) group (Figure 8). Examining, the healing process of the lesions treated with and without ASCs demonstrated that in the group with ASCs, epithelium had not regenerated completely enough to be measured. In this study, the rate of re-epithelization improved when treated in combination with CMC + ASCs. The cellular differentiation and secretion of various cytokines/parakines factors could be attributed to the mechanism through which ASCs or stem cells in general contributed towards repairing damaged tissues. Interplay between cells; that can produced VEGF, basic fibroblast growth factor (FGF) and platelet derived growth factor (PDGF-A); is responsible for the signaling cascade that accelerates the wound healing process. The early improvement of granulation tissue implies that the biological events which characterize the dynamics of wound healing were occurring more rapidly in the test group (CMC 10 mg/mL + ASCs). Cytokeratin expressions were also found to have increased significantly between control and test; indicating high biochemical and bioactive epithelial response. This pinpoints to migratory keratinocytes phenotypes and migratory epithelium, common to cutaneous wound healing response [102]. This study demonstrated that biomaterials along with specific cells which can secrete cytokines/growth factors could be the best approach for producing a graft that successfully integrates with the host tissue and prevents infection. Additionally, they also promote re-epithelization, neo-vascularization, and hair follicle formation. Current research indicates that skin regeneration highly depends upon the host cells and stem cells residing in their niche. The biomaterials only act as cell carrier, but if this biomaterial could provide the micro-environmental cue needed for dictating cellular behavior, it would provide the most suitable scenario. Biomaterials will continue to play central role in tissue engineering and regenerative medicine as they give a framework upon which dynamic tissue reconstruction could be designed. However, the more stem cells (and their molecular, genotypic and phenotypic characteristics) are explored, the better are the chances of their application; especially since stem cells hold the key to success of all tissue engineered constructs.

## 4. Conclusions

The ultimate goal of skin repair and engineering is that the skin equivalent should rapidly and completely restore the skin functions. This construct bio-functionality should also contain appendage found on normal skin, such as hair follicles, receptors, sebaceous glands, and sweat glands. Skin graft constructed using 3D printing or the hydrogel process should support rapid vascularization and facilitate nerve regeneration; only then would a graft qualify as a complete skin regenerating construct. Researchers are trying different techniques, such as reprogrammed differentiated fibroblast to attain pluripotent characteristics, thereby overcoming the time consuming step to attain the desired cell numbers from cell lines. Since these cells are similar to embryonic stem cells, they could be combined with 3D printing and help in obtaining functional tissue engineered skin. This could be a new direction to achieve biologically, structurally and functionally similar tissue to natural skin.
